# Building blocks for a public health ethics framework for the geriatric community

**DOI:** 10.4102/safp.v64i1.5414

**Published:** 2022-02-21

**Authors:** Laetus O.K. Lategan, Gert J. van Zyl, Willem H. Kruger

**Affiliations:** 1 Division of Research, Innovation and Engagement, Central University of Technology, Bloemfontein, South Africa; 2 Faculty of Health Sciences, University of the Free State, Bloemfontein, South Africa; 3 Department of Community Health, Faculty of Health Sciences, University of the Free State, Bloemfontein, South Africa

**Keywords:** care ethics, geriatric community, professional ethics, ethics, public health

## Abstract

**Background:**

The elderly population is steadily growing in South Africa. However, there is limited strategic planning or policy initiatives to address this group’s vulnerability resulting in several public health ethical issues that need to be considered and addressed. This article aims to develop a public health ethics framework for the geriatric community with the purpose to review ethical implications when working with the geriatric community.

**Methods:**

The Q-methodology was selected for data collection. Fifteen statements were ranked by means of a five-point Likert-scale questionnaire. Twenty-two participants from six geriatric institutions participated in the ranking of the statements.

**Results:**

The ranking of the statements confirmed the need for a public health ethics framework to provide guidance when working with the geriatric community and to evaluate decisions about geriatric care. Such a framework should be application-based and practice-oriented which can assist in addressing unfamiliarity with public health ethics in general and can extend the capacity for decision-making. The ranking of these statements contributed to the scope of the planned framework, by considering the vulnerability of healthcare practitioners (as community of practitioners) and the geriatric community as a basis from which to promote justice in public health programmes.

**Conclusion:**

Based on the ranking of statements, eight building blocks for a public health ethics framework were identified. The building blocks are imbedded in professional ethics and care ethics. The proposed framework can give rise to social justice in public health and the ability to evaluate what the ethical implications are for public health policies, programmes and interventions aimed at the geriatric community.

## What is the need for a public health ethics framework for the geriatric community?

Kass’^1^ seminal publication on a public health ethics framework is presented as an analytical tool to contemplate the ethical implications of proposed interventions, policy proposals, research initiatives and programmes in public health. Such a framework should enable public health workers to anticipate what the ethical implications and outcome of actions could be. This opens the possibility of alternative views on the ethical implications of public health interventions, or how ethical principles should be implemented. Any alternative should be an acceptable ethical option and not merely the satisfaction of political considerations.

Studies from Baylis, Kenny and Sherwin,^2^ Spike^3^ and Marckmann, Schmidt, Sofaer and Strech^4^ also considered what informs a public health ethics framework. Although their approaches may differ, the common agreement for such a framework lies in ethical principles relevant to public health. All three studies recommend a practice-oriented framework. From the authors’ comments, the interpretation is that a public health ethics framework needs to identify and apply ethical principles for healthcare that can promote health through healthcare services and interventions mindful of the targeted community’s needs. The aim of the public health ethics framework is not only to be ethical in healthcare delivery but also to secure that communities’ human rights are respected and advanced. The application of this framework will promote ethical healthcare delivery and add to the understanding of how to be ethical in public health.

Baylis et al.^2^ placed the emphasis on people and relationships in the context of public health’s scope. They argued that there exists the need to focus on relations amongst people instead of individuals. Their framework is influenced by relational personhood (including relational autonomy and social justice) and relational solidarity. A public health ethics framework should make the role of public health visible.

Spike^3^ took an approach of ‘mid-level’ principles. This refers to widely accepted values. Following this approach, he identified four values for public health ethics, which also have people and relationship as commonality. The principles are procedural justice (transparency of information sharing and sampling), the least restrictive alternative (upholding of human rights, least burden possible and protection of minority rights), the precautionary principle (maximum human safety) and the communitarian principle or social justice (social institutions and social connectives to individual well-being). The application of social justice is paramount in his identification of ‘mid-level’ principles.

Marckmann et al.^4^ advanced a practice-oriented public health ethics framework including both normative criteria based on an explicit ethical justification and a structured methodological approach for application. The purpose of a public health ethics framework is to provide a practical guide that can be used by public health workers. The authors opted for coherentism as a departure point. Coherentism implies that the focus is not from a single ethical principle but rather from ethical convictions and beliefs that are accepted in everyday life. A coherent framework is developed from specifying, testing and revising the commonly accepted principles into a framework for action.

The approach followed by the above authors is also reflected in the American Public Leadership Society’s *Ethical Practice of Public Health, Version 2.2*.^5^ In this code, the interdependence of people is presented as the basis for several ethical principles. As public health is directed at the health of communities, the interdependence of people is reflective of the basic aim of public health. At the same time, it is a recognition that individuals’ health is tied to their life in a community. People and relationships are, therefore, emphasised. This code identifies key public health services and accompanying ethical principles with the focus on people and relationships, more sharply defined as professional relationships. Although not explicitly mentioned, it can be observed that power domination is always a reality towards, especially, vulnerable groups.^6^

This article focuses on the geriatric community as one such vulnerable group. This group’s vulnerability is because of health, social and economic challenges.^7,8,9,10^ Such vulnerability could raise ethical dilemmas not only for the mentioned communities but also for healthcare providers. Therefore, the need for a South African public health ethics framework is based on the following arguments.

Firstly, there is a growing South African elderly population,^11,12^ but very little strategic planning or policy initiatives exist to address this group’s vulnerability, which is challenged by the impact of social determinants on their health. Service delivery and health provision are, in general, experiencing challenges as observed in the National Department of Health’s Strategic Plan, 2020–2025,^13^ and as commented on by Kelly, Mrengqwa and Geffen,^14^ Chenwi^15^ and Mathiso.^16^ Despite the growing number of people above 60 years of age in South Africa,^11,12^ corresponding with international trends,^8,10^ this remains a neglected debate in South African public health. It appears to be a similar issue in most of the sub-Saharan Africa, where there is improvement with policy formulation for healthy ageing but limited implementation of these polices.^17^

Secondly, the geriatric population analysis in South Africa suggests economic needs and health marginalisation.^18^ The reality of social isolation is reported in the literature.^14^ The question, therefore, emerges as to whether the geriatric community can stand its ground with ethical challenges either because of the way public health activities are identified and implemented or in relation to the provision of basic healthcare needs.

Thirdly, healthcare practitioners and workers delivering public health programmes may evidently not be trained sufficiently or have enough experience to consider the ethical consequences of their actions and care.^9,19^

In this article, building blocks for a framework to guide public health practitioners and workers offering care, management or administration, on how to pass ethical judgement on public health programmes and interventions aimed at the geriatric community are presented. These building blocks are application-based and practice-oriented. The framework forms part of public health ethics as a field of application that coexists alongside public health ethics as a field of study.

*Professional ethics* will be used as a basis to inform engagement with the geriatric community and *care ethics* as the ethical orientation in everyday care for the geriatric community.

## Method

### Qualitative and quantitative approach

The Q-methodology was selected for data collection and is both qualitative and quantitative in nature. The Q-methodology focuses on the subjective viewpoints of participants and is particularly useful in understanding stakeholders’ or participants’ viewpoints. This method assists in understanding how a matter is viewed and what impact these views may have during implementation.^20^ Mason^21^ associated the Q-methodology with two main stages: (1) developing the statements and sorting them, and (2) analysing and interpreting the ranked statements.

The Q-methodology outlines both consensus and deviation,^20^ which is based on ranking predefined statements (Q-sorting) relevant to the research question of a study. The statements (Q-set) are derived from the literature review and are ranked by the participants (P-set).

The advantage of this method is that it narrows many viewpoints down to a few which can be regarded as a shared way of thinking. An additional advantage is that it can include a small group (even one participant) up to a large group.

The ranking was based on presenting the statements by means of a five-point Likert-scale questionnaire (Q-sort table) where the ranking took place with 1 representing ‘least important’ to 5 representing ‘cannot do without this’. The ranking of statements can fit the two extremes of the Likert-scale, namely either agree or disagree, with a moderate or neutral point of being indecisive. The Likert-scale represents a quantitative approach within the Q-methodology.

For this article, 15 statements were identified from the literature review to develop a public health ethics framework for the geriatric community.

### Setting

Six geriatric institutions, two each in the Free State, Northern Cape and North-West provinces, were identified. These provinces have the smallest populations compared to the other six provinces and represent 29.14% of the population older than 60 years.^15^ Economically, these provinces fall outside the mainstream gross domestic product (GDP) for provinces in South Africa.^22^ The data were sampled during the time of national lockdown restrictions Level 3 (from June 2020 to 16 August 2020) and Level 2 (from 17 August 2020 to 20 September 2020) as part of the national state of disaster during the coronavirus disease 2019 (COVID-19) pandemic. Part of the restriction was that elderly people were restricted from moving out of their homes or geriatric institutions. The geriatric institutions and the participants were, therefore, identified based on the grouping of convenience sampling (most accessible environment).^23^ Purposeful sampling was also used to identify and select geriatric institutions that are in marginalised provinces and often under-serviced regions and that may not always be part of data collection on a particular topic because of their locality.^24^ The COVID-19 pandemic did not influence the collection of data as the questionnaires were delivered and collected via pre-arranged courier services.

Twenty-two participants from the six geriatric institutions participated in the ranking of the statements. Geriatric people were excluded from the study as the focus was on the gathering of in-depth information on public health ethics as perceived by the identified target population, the healthcare providers and managers. The frequency information confirmed two groups, one with medical or healthcare experience (49.9% of respondents) and one with management or administrative experience (45.4% of respondents). The information confirmed a high percentage of post-school training (72.7% of respondents), with 36.4% respondents having cumulatively 21 years and more of work experience.

### Reliability of statistics

Cronbach’s alpha was calculated to assess the reliability of the statistics. For this scale, Cronbach’s alpha is 0.912 and Cronbach’s alpha based on standardised items is 0.924. These results indicate a high level of internal consistency of the scale and unidimensionality of the items used in the scale (Table 1^25^).

**TABLE 1:** Reliability of statistics.

Cronbach’s alpha	Cronbach’s alpha based on standardised items	Number of items
0.912	0.924	15

*Source*: Lategan LOK. A public health ethics framework for the geriatric community: A South African perspective. PhD thesis in Community Health. Bloemfontein: University of the Free State 2021.

### Factor analysis

Principal component analysis (PCA) was used to summarise the information content. Based on the collected data, the rotated component matrix (Table 2^25^) identified three factors from the outcome of the survey. Variables were assigned to the factors. The rotated component matrix sorted the variables by the factor they belonged to and by co-variance with the factor.

**TABLE 2:** Rotated component matrix.

Statement	Component		
	1	2	3
Public health ethics must promote human rights in geriatric care (medical justice).	0.879	-	-
Public health ethics must promote decision-making capacity.	0.832	0.340	-
Healthcare practitioners need ethical education.	0.797	-	-
Public health ethics must protect the vulnerability of geriatric people and healthcare practitioners.	0.755	0.397	-
Leadership and management should promote ethical behaviour.	0.682	0.460	-
Ethics should be integrated in the public health ethics value chain.	0.656	-	0.656
Ethical success depends on participation around a common goal.	-	0.892	-
Healthcare practitioners need a practical guide to assist them in ethical behaviour and decision-making.	-	0.869	-
Ethical decision-making is to address the ethical dilemma at hand.	-	0.862	-
Ethics is about making a choice, implementation of the choice and evaluation of the outcome of the choice.	0.305	0.718	0.531
Ethics decision-making can have future consequences.	0.311	-	0.849
Public health policymakers do not know what the ethical needs of elderly people are.	-	-	0.734
Healthcare facilities/institutions and healthcare practitioners seldom talk about ethical challenges.	0.504	-	−0.681
Relationship building is important in ethics.	0.426	0.422	0.676
Ethics is not about who is right or wrong but about what one can do to prevent or address a moral dilemma.	0.432	0.369	0.533

*Source*: Lategan LOK. A public health ethics framework for the geriatric community: A South African perspective. PhD thesis in Community Health. Bloemfontein: University of the Free State 2021.

The PCA was used for the extraction method. For the rotation method, Varimax with Kaiser normalisation was used.

The first factor confirmed that guidelines and leadership should be evident in the application of public health ethics. This factor further confirmed that public health ethics should be part of public health practice and that ethics education is required. It is also evident that public health ethics should contribute to medical justice. Vulnerability of practitioners and the geriatric community was also confirmed.

The second factor endorsed the need for a framework to guide public health workers to make ethical decisions and evaluate the outcome of their decisions.

The third factor identified the need for a discussion on what public health ethical principles for the geriatric community are. These principles should address current moral dilemmas, prevent future dilemmas and contribute to relationship building.

## Results and discussions

The 15 statements ranked in Table 3^25^ contributed to the following results and discussions:

**TABLE 3:** Statements around a public health ethics framework.

Statement	Strongly disagree (%)	Disagree (%)	Neutral (%)	Agree (%)	Strongly agree (%)
S1: Ethical decision-making is to address the ethical dilemma at hand.	0.0	0.0	4.5	68.2	27.3
S2: Ethics is about making a choice, implementation of the choice and evaluation of the outcome of the choice.	0.0	0.0	0.0	68.2	31.8
S3: Ethics decision-making can have future consequences.	0.0	0.0	0.0	54.5	45.5
S4: Relationship building is important in ethics.	0.0	0.0	9.1	54.5	36.4
S5: Healthcare practitioners need a practical guide to assist them in ethical behaviour and decision-making.	0.0	0.0	0.0	59.1	40.9
S6: Healthcare facilities/institutions and healthcare practitioners seldom talk about ethical challenges.	4.5	4.5	31.8†	59.1	0.0
S7: Ethical success depends on participation around a common goal.	0.0	0.0	19.0	52.4	28.6
S8: Ethics is not about who is right or wrong but about what one can do to prevent or address a moral dilemma.	0.0	0.0	4.5	63.6	31.8†
S9: Public health policymakers do not know what the ethical needs of elderly people are.	0.0	0.0	27.3	40.9	31.8
S10: Healthcare practitioners need ethical education.	0.0	0.0	13.6	63.6	22.7†
S11: Leadership and management should promote ethical behaviour.	0.0	0.0	0.0	57.1	42.9
S12: Public health ethics must promote human rights in geriatric care (medical justice).	0.0	0.0	9.1	50.0	40.9
S13: Public health ethics must protect the vulnerability of geriatric people and healthcare practitioners.	0.0	0.0	4.5	50.0	45.5
S14: Public health ethics must promote decision-making capacity.	0.0	0.0	9.1	54.5	36.4
S15: Ethics should be integrated in the public health ethics value chain.	0.0	0.0	0.0	54.5	45.5

*Source*: Lategan LOK. A public health ethics framework for the geriatric community: A South African perspective. PhD thesis in Community Health. Bloemfontein: University of the Free State 2021.

† , The percentages in the table are rounded up to first decimal place.

The ranking of statements presents the following results which are discussed either per ranking of statement or groups of statements depending on their interrelatedness.

The ranking of statements presented only one absolute strongly disagree ranking to the value of 4.5% (Statement 6). Only one strongly agree category was not ranked (Statement 6). The agree ranking for this statement was slightly below 60.0%. For 14 statements, the agree and strongly agree rankings are high in total, ranging from 70.0% to 100.0%. The ranking pattern can, therefore, be interpreted as in agreement with the need for a public health ethics framework for the geriatric community. This interpretation is further supported by no ranking of disagree or strongly disagree categories except for Statement 6. This added to the confirmation of the need for a public health ethics framework for the geriatric community.

The ranking of Statement 6 followed an interesting pattern if evaluated against the ranking of the other statements. This is the only statement that excluded the strongly agree ranking. The absence of a strongly agree ranking indicated that no respondent regarded this as a statement that they cannot do without. The rankings also illustrated the variance in opinion. The 31.8% neutrality statement can be interpreted in different ways. First, the wording of the neutral as the midpoint suggested that there is no specific view on this matter, either because of unfamiliarity with the topic, or because the topic is ambiguous or socially not desirable. Second, neutral should not be confused with undecided or do not know. Third, the neutral scale is the easiest way to respond to a question.^26^ Given the content of the statement, the reasonable interpretation is that the midpoint response is because of ignorance. This interpretation can be supported by the 9.0% disagree or strongly disagree and 59.1% agree rankings. The contribution of this statement’s ranking to the planned framework pointed towards the level of familiarity of healthcare workers with the ethical challenges of the geriatric community.

Within the ranking of the statements, 67.0% of them had at least one respondent using the neutral ranking for the statement. The Likert scale used for ranking these statements was an interval scale using equal intervals as opposed to an ordinal scale using rank-ordered levels. In view of the above explanation of the neutral ranking, the interpretation is that the respondents may not be familiar with the topic. The neutral rankings of Statements 6, 7 and 9 with 31.8%, 19.0% and 27.3%, respectively, confirmed possible ignorance on the content of the statements. These statements, however, were focused on the familiarity of public health ethics and not on the desire to not have such a framework.

In Statement 5, there was a 100.0% agree or strongly agree ranking in support of a practical guide to assist healthcare practitioners in ethical behaviour and decision-making. This was further supported by the 100.0% agree or strongly agree ranking in Statement 15 that ethical principles cannot be selectively captured in this framework. The need for such a practical guide was further supported by the 90.1% ranking of Statement 14 that the framework should promote decision-making capacity. The agree ranking of 59.1% of Statement 6 suggested that there may not be sufficient discussion of ethics in a particular geriatric institution, or that some healthcare workers may have been left out or were not aware of this discussion. A similar observation followed from Statement 9. The 72.7% agree or strongly agree ranking implied that the ethical needs of geriatric people may not be known to healthcare practitioners. Based on the coding scheme and data, this observation appears to be common across participating institutions.

Either way, these statements underlined the need for such a guide. Based on the ranking in Statement 4, the 90.1% agree or strongly agree ranking implied that such a framework should contribute to more than merely what ethics is as a theory or description (Statements 1 & 2) or to make decisions (Statement 14). The framework should also contribute towards creating relationships (Statement 4) and preventing or addressing moral dilemmas (Statement 8). Statements 4 and 8 have an agree or strongly agree ranking higher than 90.0%. The ranking of Statements 1 and 2 is in line with what is generally accepted as ethics or the scope of ethics. From these statements, the conclusion is that there exists a need for a public health ethics framework.

Statement 13 captured the changed perspective from vulnerability of the healthcare receiver only, to also acknowledge the vulnerability of the healthcare provider. In the context of this study, there is a 95.5% acknowledgement that healthcare practitioners are vulnerable too, although presumably for different reasons. This acknowledgement is important in relationship building and advancement of ethical public values. Statement 12 had a 90.9% agree or strongly agree ranking that human rights in healthcare should be promoted. This positive view on human rights promotion and the shared purpose of ethics confirms what the purpose and content of such a framework should be. Based on the coding scheme and data, the neutrality ranking was not representative of all participating institutions. The ranking of these statements contributed to the scope of the planned framework to consider the vulnerability of healthcare practitioners (as community of practitioners) and the geriatric community as the basis to promote justice in public health programmes and interventions.

The scope of a public health ethics framework is further informed by Statements 2 and 3. Both statements have a 100.0% agree or strongly agree ranking that ethics is more than just knowing what the principles for public health ethics are. Statement 2 reflected on decision-making, decision implementing and evaluating the outcome of the decision. This statement consolidated that ethics has principles (theory basis of ethics) that must be applied to a situation (practice of ethics). Statement 8 contributed to the interpretation of this ranking. The 94.4% agree or strongly agree ranking of this statement suggested that knowing public health principles is not enough as the significance of ethics is to address or prevent ethical dilemmas in public health. Statement 3 contributed to an important matter in ethics, namely that ethical applications may *now* be the best for the situation in hand, but the application may lead (later) to another ethical challenge. With this comment, the universality of an ethical principle is confirmed but applications are unique to a situation and may not be applicable without revision for future similar situations. The meaning of these statements for the planned framework is that ethical principles can be identified but their applications for all possible scenarios cannot be identified.

Statement 9 highlighted an important requirement in public health ethics, namely, to know the ethical needs of the geriatric community that public health workers are working with. The agree or strongly agree ranking of 72.7% suggested that this knowledge is absent. The 27.3% neutrality statement indicated that this is not a shared perspective. The ranking groups (agree or strongly agree and neutral) invited the question of whether ethical needs were not read as needs only. However, the value of this statement is that a framework is based on the general assumption that public health practitioners are familiar with the ethical needs of elderly people. This assumption is further aligned with Statement 6. Although there was a 59.1% agree ranking of this statement, the 9.0% disagree and strongly disagree ranking represented a different opinion. Although the neutral ranking of 31.0% cannot be read together with the disagree or strongly agree rankings, it is evident that talks about ethical challenges are certainly neither absent nor active.^27^

Statement 11 had a joint 100.0% agree and strongly agree ranking that leadership and management should promote ethical behaviour. The responsibility goes further beyond managers only as Statement 10 indicated an 86.3% need for ethics education. The two statements supported the ethical responsibility of all working with the geriatric community, regardless of a person’s responsibility. This interpretation can be read together with Statement 7 that ranked ethical success based on a common goal with a joint 81.0% agree or strongly agree ranking.

### Development of the building blocks for a public health ethics framework

The ranking of Statements (1, 2, 5, 11, 14 & 15) confirmed the need for a public health ethics framework for the geriatric community that can be used in geriatric facilities and institutions. The statements further suggest the purpose of such a framework, namely, to create knowledge, empower decision-making on applying ethical principles to public health and to consider the consequences of applying ethical principles.

In drafting this framework, the approach suggested by Marckmann et al.^4^ and Spike^3^ is followed. Based on their recommendations, such a framework must be developed from what ethical principles are known, or from what are the most common ethical principles associated with public health for the geriatric community. The leading principle for all ethics is taken as ‘do no harm’.^1^

Working with the geriatric community, the ethical principles of (1) respecting their vulnerability and fragility, (2) protecting their lives from abuse and neglect, and upholding dignity, (3) securing a safe environment to live in, and (4) providing quality access to healthcare and provision, can be regarded as the ethical basis of public health for the geriatric community.^28,29,30,31^

The 90.0% agree or strongly agree ranking of Statements 8, 12 and 13 substantiates the basis of understanding of public health ethics for the geriatric community. The ranking of the statements further suggests that although important, public health ethics is in scope different from medical ethics dealing with doctor-patient relationships and bioethics dealing with matters around life and death. The significance in outlining the conceptual difference between public health ethics, medical ethics and bioethics, accents the compelling need to know what the purpose of public health is, and what ethics foundation is required for this provision. A framework for public health ethics departs, therefore, from what public health is and what ethical principles should accompany public health. This represents a typical applied ethics approach to public health.^2^

The ethical principles outlined above are not limited to the physical and mental conditions of geriatric population only, but also include the environment in which people live and how services are presented to them. During the COVID-19 pandemic, the shift from an individual, patient centred medical ethics towards a public health ethics was evident.^32^ Public health interventions such as lockdown, quarantine and compulsory mask-wearing shifted the focus from individual patients to the collective. Public health ethics has at its core the welfare of the majority in society. The primary task of public health ethics is the allocation of resources in such a way that it maximises population health. Transparency is key to a public health ethics framework. It is for this reason that public health ethics is utilitarian in nature.

Based on these comments, the *first building block* for this framework is promoting the core value of public health.

The *second building block* is to identify the ethical principles for public health from the core value of public health.

Applying this view to the planned framework, the next step would be to acknowledge that public health workers (as agents) and the geriatric community (as recipients of public health programmes) are vulnerable for their own reasons as identified by Statement 13. Where the vulnerability of the geriatric community is primarily linked to geriatric people’s health, social and economic needs, less is known about the vulnerability of healthcare providers. Without ignoring their vulnerability for most probably the same reasons as above, workplace challenges can contribute to their vulnerability. Reports on corruption related to COVID-19 procurement, and the difficulty of rolling out the vaccine programme for the geriatric community, support the observations of workplace challenges.^33^ The fact that the National Department of Health of South Africa does not have a strategic plan to address the needs of a growing geriatric community confirms the under-preparedness for this development.^11^ The point in this case is that the workplace can contribute to the ethical challenges experienced by healthcare practitioners.

The *third building block* for this framework is recognising ethical challenges for the agent and recipient of service.

Statements 10 and 11 identified the need for education, leadership and management. The expectation is that there should be an in-service training programme for healthcare practitioners. Ethical leadership is not unfamiliar with ethics. Ethical leadership has honesty, transparency, impartiality and integrity as trademarks. Of note is the comment that ethical leaders lead ‘from a sense of duty, not politics’.^34^

The *fourth building block* for this framework is to advance ethical leadership.

With regard to ethics training, a familiar approach is to start from an *inside-in* approach where the existing knowledge foundation of ethics is taken as the basis for training. This seems to be more effective than to start from an *outside-in* approach where ethics is first explained. Such a procedure promotes tacit learning. It will also avoid a narrow understanding of ethics as theory only and will promote the application of the principles associated with ethics for the geriatric community.^35^

The *fifth building block* for this framework is to introduce ethics education.

Because the focus is on public health ethics, the South African Constitution’s right of access to healthcare can be the focal point in education. Horn^36^ correctly linked public health with social justice as more than just beneficence is required in public health. Although her proposal is in the context of global healthcare and moral cosmopolitanism, the tipping point is that the practice of an ethics-based public health system includes much more than merely goodness or kindness to people. Her advocacy can be expanded to medical justice as expression of social justice in healthcare. This concept of ‘medical justice’ is most appropriate to public health ethics. Although this concept is more familiar in medical insurance claims and treatment of patients, the essence of medical justice is to protect receivers and providers of healthcare from bad practices, and to ensure reliability in interpreting standards of care.^37^ Equity, access to resources and services, participation and rights are commonly regarded as the core values of social or medical justice in healthcare. Wallack^38^ remarked that public health challenges originate from injustice and inequality in society. For effective public health delivery, a narrative should be developed that communicates social justice values. The acceptance thereof will contribute to translating values in caring and formulating effective public policy. These remarks instil the importance of shared responsibility.^34^

The *sixth building block* for this framework is promoting social justice in public health programmes, interventions and delivery.

This framework is imbedded in (1) professional ethics and (2) care ethics.

Professional ethics is based on ethical principles for the workplace. It guides the behaviour between the agent and recipient of service. Professional ethics emerges from the professional engagement between people in a concrete situation. Professional ethics can be captured in a professional code which is typically deontological in nature. Professional codes can be linked to a profession, an organisation or a sector. Verbruggen^39^ argued for an ethical professional expertise that is vested in a communicative ethics. This requires a dialogue amongst professionals on the ethical principles for their profession. An approach where work expertise is imbedded in ethical principles may be more conducive considering different levels of training, experience and responsibly in the workplace.

The *seventh building block* for this framework is developing ethical expertise.

Care ethics originates from a relationship to address responsibility, vulnerability and power challenges between the caregiver and care-receiver and all who are engaged in care. Care ethics is practice-orientated and deals with ethical challenges in daily care.^40^ What can be added is that care ethics is directed at the uniqueness and specificity of the situation and not at a common rule or value.^41^ Applied to public health and geriatric communities, care ethics is the *relationship* between public health agency and geriatric community, recognising their mutual vulnerability and potential power relationship within healthcare provision and carrying out the *responsibility* to improve healthcare services and provision.

The *eighth building block* for this framework is practising care ethics.

The suggested building blocks for a public health ethics framework is presented in Table 4.^25^

**TABLE 4:** Building blocks for a public health ethics framework.

Building block	Focus of building block	Benefit of building block
Promote the core value of public health.	Communities and not an individual only.	Community centred within promotion of health and prevention of disease.
Identify the principles for public health ethics.	Promotion of health, respect for life, dignity and vulnerability, quality of living and environment and access to services.	Ethical awareness in public health for the geriatric community.
Recognise ethical challenges for the agent and recipient of service.	Ethical challenges in the workplace and community.	Awareness of ethical challenges and vulnerability.
Advance ethical leadership.	The practice and promotion of ethics.	Ethical behaviour between the healthcare provider and the healthcare recipient.
Introduce ethics education.	Knowledge of/about ethics.	Preparedness to evaluate and deal with ethical challenges.
Promote social justice.	Equality of public health policies, programmes and interventions.	Equity and equality in public health.
Develop ethical expertise.	Practice-oriented understanding of ethics in public health.	Professional behaviour.
Practise care ethics.	Relationship building.	Avoid power domination towards vulnerable groups.

*Source*: Lategan LOK. A public health ethics framework for the geriatric community: A South African perspective. PhD thesis in Community Health. Bloemfontein: University of the Free State 2021.

## Conclusion

The 15 statements ranked in search of a public health ethics framework confirmed the undisputable need for such a framework. In addition, the need was expressed that it should be a user-friendly framework, steering away from a theoretical understanding of ethics to an application-based and practice-oriented understanding of ethics. This framework can promote social justice in public health and the ability to evaluate what the ethical implications are for public health policies, programmes and interventions aimed at geriatric communities.

Eight building blocks were identified for the framework.
